# Behavior of *Salmonella* Typhimurium on Fresh Strawberries Under Different Storage Temperatures and Wash Treatments

**DOI:** 10.3389/fmicb.2018.02091

**Published:** 2018-09-13

**Authors:** Wen Wang, Yu Zhou, Xingning Xiao, Guiling Yang, Qiang Wang, Wei Wei, Yuanjing Liu, Hua Yang

**Affiliations:** ^1^MOA Laboratory of Quality and Safety Risk Assessment for Agro-Products, State Key Laboratory Breeding Base for Zhejiang Sustainable Plant Pest Control, Institute of Quality and Standard for Agro-products, Zhejiang Academy of Agricultural Sciences, Hangzhou, China; ^2^State Key Laboratory of Tea Plant Biology and Utilization, School of Tea and Food Science and Technology, Anhui Agricultural University, Hefei, China; ^3^College of Biosystems Engineering and Food Science, Zhejiang University, Hangzhou, China

**Keywords:** *Salmonella* Typhimurium, strawberry, storage, wash, modeling

## Abstract

Fresh strawberries are one of the most popular fruits in China and are vulnerable to microbial contamination. In this study, the behavior of *Salmonella* Typhimurium on fresh strawberries stored at refrigeration and room temperatures, as well as the effectiveness of mild heat wash treatments at 47, 50, and 53°C on bacterial survival was investigated. The modified Gompertz, Huang, log-linear, and Weibull models were used to fit bacterial growth and survival curves under different treatments. A secondary model based on linear regression was developed to describe the effect of washing temperature on the kinetic parameters of *S*. Typhimurium survival derived from the Weibull model. During 72 h storage, *S*. Typhimurium on fresh strawberries stored at 4°C was reduced by 1.35 log CFU/g and growth of 5.64 log CFU/g was observed when strawberries were stored at 25°C. Bacterial reductions of 1.22 ± 0.15, 1.92 ± 0.06, 2.27 ± 0.07 log CFU/g were obtained when washing was carried out at 47, 50 and 53°C for 240 s, respectively. The wash temperature was an important parameter for bacterial inactivation and bacterial populations declined significantly in conjunction with washing time (*p* < 0.05). Warm wash treatments lead the visible color changes of strawberries, showing a slightly darker appearance while acceptable. The goodness-of-fit indices indicated that the log-linear model provided a satisfactory fit to describe the bacterial survival at 4°C. According to the smaller Akaike information criterion (AIC) value, the modified Gompertz model performed slightly better than the Huang model in describing bacterial growth at 25°C. The high *adj*-R^2^ (≥0.90) and small RMSE (≤0.22) indicated the Weibull model better described bacterial behavior under mild heat treatments. We found a close linear relationship between wash temperatures and ln *k* and ln *n*. These models were validated by independent experimental data and the values of the bias and accuracy factors fell into the acceptable range.

## Introduction

*Salmonella* is a leading cause of global foodborne illness and in China accounts for 70 to 80% of foodborne pathogenic outbreaks (Wang et al., 2007; Scallan et al., 2011; Shao et al., 2011). There are >2,600 *Salmonella* serotypes and the *Salmonella enterica* serotypes Enteritidis, Typhimurium, Newport, Javiana and Heidelberg are the most common (Guibourdenche et al., 2010; Reddy et al., 2016). Although *Salmonella* is commonly associated with meat contamination, it is also linked to fresh produce (Sivapalasingam et al., 2004). Between 1998 and 2008, fresh produce contamination amounted to 46% of annual foodborne illnesses in the United States and 38% required hospitalization and 23% resulted in death (Painter et al., 2013). However, food safety data regarding *Salmonella* on fresh produce and especially fruit in developing countries is very limited (Gomba et al., 2016).

Strawberries (*Fragaria* × *ananassa*) are in high consumer demand, are rich in vitamin C, iron, potassium and fiber, and may have anti-cancer properties (Stoner et al., 1999; Mireku, 2012). In China, strawberry production has increased rapidly and China is now a major strawberry producer with a total planting area of 130,000 ha and a production of 3.5 million tons per year (Ministry of Agriculture of China, 2017). Benihoppe (Akihime × Sachinoka) is the most popular strawberry cultivar in China with a high sugar and a moderate acid content coupled with excellent flavor (Mireku, 2012). Although berries are low-risk foods for bacterial contamination due to their naturally low pH, strawberries are field-packed and sold unwashed. Therefore, there is the possibility for microbial contamination during cultivation, harvest and postharvest handling (Knudsen et al., 2001; Sreedharan et al., 2015). Poor worker hygiene, contaminated irrigation water and close association with soil are the most likely sources of pathogen contamination on strawberries. Frozen strawberries have been linked to three outbreaks of hepatitis A (Dougherty et al., 1965; Niu et al., 1992; Hutin et al., 1999). A recent study revealed that 1/143 of imported strawberries in the US was contaminated with *Salmonella* (Food and Drug Administration, 2001). More recently, strawberries have been associated with a bacterial foodborne illness outbreak (Food and Drug Administration, 2011). Although there was rarely strawberry related outbreak reported in China, pathogens, including *Salmonella, Staphylococcus aureus* and *Enterobacter cloacae* have been identified in previous studies, posing a potential risk to public health (Pan et al., 2007; Dai et al., 2017). In spite of these risks, strawberries are not washed or disinfected prior to delivery to market because these practices have a negative effect on flavor. Therefore, it is important to identify the optimal handling practices to minimize the threat posed by *Salmonella* and other pathogens.

Current food safety objectives emphasize that increasing consumer self-protective behaviors is the most direct method to limit illness due to contamination (Codex Alimentaris Committee, 2004). According to our preliminary survey of household consumer practices, almost half of the responses stated that they leave strawberries at room temperature and 16% do not wash them before consumption (data not shown). However, there is little information concerning the pathogen behavior on fresh strawberry under different storage temperatures and wash treatments, which is critical information for quantitative microbial risk assessment and data-based decision. Therefore, the objectives of this study were (i) to determine *Salmonella* Typhimurium survival and growth when inoculated onto strawberries under different storage temperatures (ii) to evaluate the bacterial inactivation effect of wash treatments and (iii) to use mathematical models to describe the bacterial behaviors. Our findings will help identify conditions favorable for the safe storage and washing of strawberries in the household. This study also provides information for a quantitative risk assessment model of *Salmonella* on strawberries.

## Materials and methods

### Bacterial strains

The *S*. Typhimurium strains used in this study were ATCC 14038 (American Type Culture Collection, Manassas, VA, USA) and S9 (a strain isolated from chickens in our laboratory). The bacteria were stored at −80°C in Luria-Bertani broth (LB, Difco, Franklin Lakes, NJ, USA) containing 20% (v/v) glycerol. Each strain was separately incubated in LB broth at 37°C for 24 h. Equal volumes of each suspension culture were combined to obtain a two-strain mixture of *S*. Typhimurium before inoculation.

### Strawberry samples and inoculation

Fresh strawberry samples (*Fragaria* × *ananassa* Dutch, Benihoppe; pH ~ 4.0) were purchased from a local supermarket the day before each experiment. Samples with visible physical injuries were discarded and only intact, healthy and ripe samples with similar size and weight (approximately 7–8 g) were selected. Strawberry stems were removed and the samples were washed with sterile distilled water and dried for 60 min in a laminar-flow hood at room temperature to remove excessive moisture. Bacterial mixtures were diluted to appropriate concentrations for sample inoculation. In contrast to the dipping method, our spot inoculation method more closely mimics bacterial contamination that might result from contact with contaminated hands, animal feces or soil (Knudsen et al., 2001). Therefore, each strawberry sample was placed on a flat surface and bacterial suspensions were dripped onto the intact surfaces. The samples were then held at room temperature for 30 min under the laminar-flow hood to allow bacterial attachment. Samples were analyzed to determine the initial inoculation level before storage or washing.

### Storage and wash treatment of strawberry samples

Inoculated strawberry samples were placed in food preservation packages and stored under refrigeration (4°C) and at room temperature (25°C) for 72 h in an incubator (Binder, Tuttlingen, DE). Every 8 h, samples were transferred to individual sterile stomacher bags (177 by 305 mm; Seward, London, UK) containing 20 ml of buffered peptone water (Becton Dickenson, Sparks, MD, USA) and homogenized for 2 min using a food stomacher instrument (Model 400, Seward) to determine the bacterial population numbers. For the wash treatment, inoculated strawberry samples were submerged in a sterile stainless steel vessel containing 500 mL of sterile distilled water. The vessels were placed in a bath chamber at the target temperatures (47, 50, and 53°C), and washed for 240 s while rotating at 90 rpm. A data acquisition system (Model 34970A, Agilent, Santa Clara, CA) was used to monitor the temperature with three K-type thermocouples inserted into the bath chamber and the vessel at different locations. The range of temperatures and washing times were chosen based on consumer surveys and our preliminary experiments. The rotation represented a household situation where the strawberries could be hand-shaken in a container for a few minutes. Bacterial populations were determined every 40 s. Each datum point of bacterial growth and survival represented an average of three individual experiments in which each treatment contained two samples (*n* = 6).

### Bacterial enumeration

Bacterial populations on strawberries were examined using the spiral plating method. The homogenates were serially diluted 10-fold in buffered peptone water and appropriate dilutions were automatically spread on the xylose lysine deoxycholate agar plates (Becton Dickinson) in duplicate using a spiral plater (WASP 2, Don Whitley, Shipley, UK). The plates were incubated at 37°C for 16–18 h and colonies were enumerated using a ProtoCOL automated colony counter (Synbiosis, Cambridge, UK). The limit of detection was one colony for each 50 μL sample (~60 CFU/g).

### Mathematical models

The growth/survival patterns of *S*. Typhimurium under different storage temperatures were plotted as the log of the population size (log *N*_*t*_) against time (*t*), and were analyzed to develop primary models. Two primary models were chosen to fit the growth data collected from the room temperature storage experiment, and their performances were compared in determine the model with the best fit. The first model was the modified Gompertz (Equation 1), which has been widely used in bacterial growth modeling studies (Zwietering et al., 1990).

(1) $$\begin{array}{l} \text{log }N_{t}=\text{log }N_{0}+(\text{log }N_{\max}-\text{log }N_{0})\\ \;\exp\left\{-\exp\left[\frac{\mu_{\max}e}{\text{log }N_{\max}-\text{log }N_{0}}(\lambda -t)+1\right]\right\} \end{array}$$

The second primary model was the Huang (Equation 2) (Huang, 2013), which is especially suitable for growth curves with three phases (lag, exponential, and stationary).

(2) $$\begin{array}{l} \text{log }N_{t}=\text{log }N_{0}+\text{log }N_{\max}-\ln\\ \left\{e^{\text{log }N_{0}}+\left[e^{\text{log }N_{\max}}-e^{\text{log }N_{0}}\right]e^{-\mu_{\max}B(t)}\right\}\\ \;B(t)=t+\frac{1}{\alpha }\ln\frac{1+e^{-\alpha (t-\lambda )}}{1+e^{\alpha \lambda }} \end{array}$$

(3) $$B(t)=t+\frac{1}{\alpha }\ln\frac{1+e^{-\alpha (t-\lambda )}}{1+e^{\alpha \lambda }}$$

In Equations 1–3, *N*_0_, *N*_*max*_, and *N*_*t*_ (CFU/g) represent the initial, maximum, and time *t* (h) bacterial populations, respectively; λ is the lag time (h); μ_max_ is the specific growth rate (h^−1^); the lag phase transition coefficient α is 4.0 in the Huang model (Huang, 2013).

For the survival curve of *S*. Typhimurium under refrigeration, we used a log-linear model. This model assumes a homogeneous bacterial resistance to refrigeration described with a linear relationship between the log of the population density and storage time. It has been frequently used for bacterial survival curve fitting (Lori et al., 2007; Li et al., 2013; Huang, 2014).

(4) $$\text{log }N_{t}=\text{log }N_{0}-\frac{t}{D}$$

where *N*_0_ and *N*_*t*_ (CFU/g) are bacterial populations at the initial and time *t* (s), and *D* is the decimal reduction time (s).

The bacterial inactivation curves by wash treatments were fitted with the Weibull model based on the assumption that the bacterial resistance to heat treatment varies from one cell to the other and follows a Weibull distribution (Peleg and Cole, 1998). This model is simple and flexible (Equation 5).

(5) $$\text{log }N_{t}=\text{log }N_{0}-k\times t^{n}$$

where *N*_0_ and *N*_*t*_ (CFU/g) are initial population numbers and time *t* (s); *k* is the scale parameter of the model and *n* is the shape parameter that depicts the shape of the bacterial survival curve. For *n* = 1, a linear curve is obtained, *n* > 1 describes a convex curve (shoulder) and indicates that the remaining cells are dying, whereas *n* < 1 shows a concave curve (tailing) and indicates that the remaining cells have the ability to adapt to the applied stress (Wang et al., 2013).

A secondary model was generated using linear regression to describe the effect of temperature on the parameters *k* and *n*. A natural logarithmic transformation was performed to homogenize the variance of *k* and *n*.

(6) $$\text{ln }k=a_{0}+a_{1}T$$

(7) $$\text{ln }n=b_{0}+b_{1}T$$

where T is washing temperature (°C), and *a*_0_, *a*_1_, *b*_0_ and *b*_1_ are the regression parameters of the model.

### Model evaluation and validation

The goodness-of-fit of primary models were evaluated by the *adj-R*^2^ and root mean square error (RMSE). Akaike information criterion (AIC) values were calculated for comparing models (Akaike, 1981; Burnham and Anderson, 2002). For each set of experimental data, the AIC is given by:

(8) $$AIC=2k-2\ln(L)=2k+n\left[\text{ ln}(\frac{2\pi \times RSS}{n})\right]+1$$

where *k* is the number of parameters in the model, *n* is the number of data points on the survival curves, *L* is the maximum value of the likelihood function and *RSS* is the residual sum of squares in the model. The model with the minimum AIC value is preferred to fit the data (López et al., 2004; Li et al., 2013).

In addition, models were externally validated by independent trials under different experimental conditions within the model ranges but were not included in model establishment. The indices of bias factors (*B*_*f*_) and accuracy factors (*A*_*f*_) were calculated accord to Ross (Ross, 1996).

(9) $$B_{f}=10^{\left[\sum_{i=1}^{n}\text{log }(\frac{obs}{pred})/n\right]}$$

(10) $$A_{f}=10^{\left[\sum_{i=1}^{n}\left|\text{log }(\frac{obs}{pred})\right|/n\right]}$$

where *n* is the number of trials, *obs* is the observed values from the independent experiments and *pred* is the predicted values calculated by the developed models. The *B*_*f*_ shows the structural deviation, indicating that a model either overpredicts (*B*_*f*_ > 1) or underpredicts (*B*_*f*_ < 1) the observed values. *A*_*f*_ indicates the difference between observations and predictions.

### Color measurement

CIE (International Commission on Illumination) parameter was considered to quantify the color of the sample in a three dimensional domain where *L* (lightness), *a* (redness) and *b* (yellowness) are the corresponding mutually perpendicular axes (Robertson, 1977). The values were determined by placing the probe of the colorimeter Chroma Meter CR 400 (Minolta Osaka, Japan) onto the strawberry surface. The total color difference (Δ*E*) was calculated according to Equation (11). Depending on the value of Δ*E*, the color difference between the treated and untreated samples could be estimated such as not noticeable (0–0.5), slightly noticeable (0.5–1.5), noticeable (1.5–3.0), well visible (3.0–6.0) and great (6.0–12.0) (Cserhalmi et al., 2006). All measurements were taken on three sites of each washed sample.

(11) $$\Delta E=\sqrt{\Delta L^{2}+\Delta a^{2}+\Delta b^{2}}$$

where Δ*L*, Δ*a*, and Δ*b* are the differences of lightness, redness, and yellowness between the treated and untreated samples, respectively.

### Curve fitting and statistical analysis

The bacterial counts were analyzed by calculating the means (*n* = 6) and standard deviations (SD) using Excel 2010 (Microsoft Corp., Redmond, WA). The bacterial growth and survival curves obtained were fitted with the USDA Integrated Pathogen Modeling Program (IPMP 2013) that allows users to analyze kinetic data without the need for programming (Huang, 2014). OriginPro 8.1 software (OriginLab, Northampton, MA, USA) was used for modeling the bacterial inactivation under wash treatments. Analysis of variance (ANOVA) was performed using the least squares techniques and the significance of the differences was established at *p* < 0.05.

## Results and discussions

### Bacterial growth and survival on strawberries at different storage temperatures

**Figure 1:**
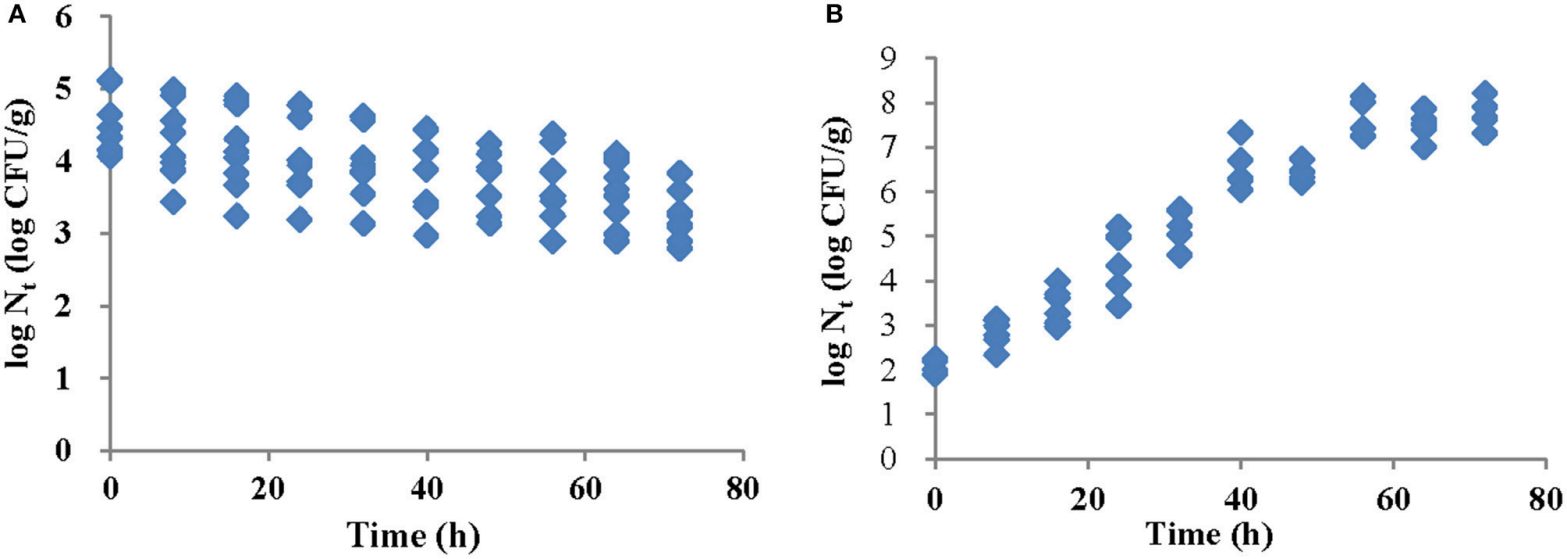
Changes in *S*. Typhimurium populations on strawberries after 72 h at **(A)** 4°C and **(B)** 25°C.

*Salmonella* Typhimurium growth and survival on strawberry samples was evaluated every 8 h during storage under refrigeration (4°C) and at ambient temperature (25°C). Initial inoculation levels on strawberry samples were 4.56 ± 0.50 log CFU/g and 2.13 ± 0.15 log CFU/g for refrigeration and ambient temperature storage, respectively. Changes in bacterial populations over 72 h are shown in Figures 1A,B. There was a significant bacterial decline (approximately 1.35 log CFU/g, *p* < 0.05) on strawberries stored at 4°C. At 25°C, the *Salmonella* population increased to 7.77 log CFU/g after 72 h. Visual inspection during the storage did not reveal the occurrence of mold or decay at either temperature.

The survival of *S*. Typhimurium on intact strawberries under refrigerated storage was similar to that previously reported for *Salmonella* spp. and other pathogens (Knudsen et al., 2001; Yu et al., 2001; Flessa et al., 2005). When strawberries were stored at 5°C, populations of *Salmonella* spp. and *E. coli* O157:H7 decreased by 1–2 logs on the surfaces of intact strawberries at 3 days and remained constant on cut surfaces over 7 days. This suggested that the higher humidity on cut strawberries would enhance bacteria survival (Knudsen et al., 2001). A significant reduction of *E. coli* O157:H7 (1.3–2.25 log CFU/g) on strawberry surfaces was found after 3 days under refrigeration storage (Yu et al., 2001), However, bacteria inside the fruit survived and surface survival may be a result of desiccation, nutrient deprivation or growth competition. A 3-log reduction of *Listeria monocytogenes* over a 7-day storage period was found in intact strawberries while the populations on cut surfaces remained constant (Flessa et al., 2005). *Salmonella* spp. populations on fresh-cut pineapple declined slightly but increased 0.5–1 log CFU/g on fresh-cut honeydew melons, cantaloupes, watermelons, pitayas, mangos and papaya after 6 days (Feng et al., 2017). These studies demonstrated that the soluble solid content and humidity on cut surfaces could provide adequate nutrition to enhance the bacterial survival or growth. Therefore, choosing intact strawberries and avoiding mechanical injury even under refrigeration storage should be emphasized for consumer education.

*Salmonella* is capable of significant growth on fresh-cut fruits stored at room temperature. For example, *Salmonella spp*. on fresh-cut watermelons and papayas reached 8.0 log CFU/g after 2 days at 20°C (Penteado and Leitão, 2004). Similarly, *Salmonella spp*. on fresh-cut honeydew melons, cantaloupes, watermelons, pitayas, mangos and papaya stored at 25°C could reach 8.0 log CFU/g after 3 days (Feng et al., 2017). We found that *S*. Typhimurium levels on intact strawberries increased from 2 to 7.7 log CFU/g when stored at 25°C for 3 days. The acidic nature of fruits including strawberries, mangoes and pineapples are not the only consideration in *Salmonella* growth inhibition (Knudsen et al., 2001; Ma et al., 2016). *Salmonella* can grow at pH 4 and the grow-limiting pH levels vary with acid type, incubation temperature, inoculum level and relative oxygen supply (Chung and Goepfert, 1970). A previous study using 13 *Salmonella* strains in tryptone-yeast extract-glucose medium acidified with HCl (pH 3.8 to 4.0) at 30°C reported substantial bacterial growth in 2–3 days (Ferreira and Lund, 1987). Tomatoes can also provide a favorable environment for *Salmonella* growth in spite of their low pH, which is most likely the result of the high (80%) citric acid content (Asplund and Nurmi, 1991, Beuchat and Mann, 2008). In addition, pineapples were unable to support the growth of acid-adapted *Salmonella* strains due to the acid type, concentration, the presence of non-fermentable fiber and the lack of nutrients (Strawn and Danyluk, 2010a; Abadias et al., 2012). Acid tolerance appears to be common among *Salmonella* serovars and *S*. Typhimurium can grow in apple juice (pH 3.4 to 3.9), *Salmonella* Chester on apple disks (pH 4.1) and *Salmonella* Montevideo in tomato core tissue (pH 4.1 to 4.3) (Goverd et al., 1979; Wei et al., 1995; Zhuang et al., 1995; Liao and Sapers, 2000). The bacterial responses to stressors such as the low pH of fresh cut mango surfaces can contribute to *Salmonella* growth at 23°C regardless of initial population concentrations (Foster and Spector, 1995; Strawn and Danyluk, 2010b). Thus, *S*. Typhimurium growth on intact strawberries that we observed indicates the importance of refrigeration to suppress growth.

### Bacterial inactivation during washing and color changes of treated strawberry

We used an initial *S*. Typhimurium inoculation level on strawberry samples for wash treatment at 3 log CFU/g. Our preliminary tests revealed that the reduction in bacterial levels was modest with a reduction of 0.20–0.38 log CFU/g after washing at 25°C for 240 s, and there was no significant effect of washing time on the bacterial reduction (*p* > 0.05). However, we found a greater reduction when the washes were carried out with warm water. Levels were reduced by 1.22 ± 0.15, 1.92 ± 0.06, 2.27 ± 0.07 log CFU/g using temperatures of 47, 50, and 53°C, respectively, for 240 s (**Figure 4**). Bacterial populations declined significantly with washing time and temperature (*p* < 0.05). Therefore, household washing is a critical step to control microbial contamination on ready-to-eat produce.

The CIE parameters of the unwashed strawberries were *L* = 35.66 ± 0.82, *a* = 36.39 ± 0.50, and *b* = 16.29 ± 1.25. The values of lightness, redness and yellowness of the strawberries decreased after most wash treatments (*p* < 0.05). The total color difference (Δ*E*) of different temperature-time wash treatments of strawberries were showed in Figure 5. The changes of color were < 3 and not statistically different for washing time < 80 s at 47, 50, and 53°C (*p* > 0.05). The maximum Δ*E* was around 5.4 obtained at washing time of 240 s at 50 and 53°C. Since Δ*E* was considered as “not noticeable,” “slightly noticeable,” “noticeable,” “well visible,” and “great” when it crosses the corresponding lower limit of 0, 0.5, 1.5, 3, and 6, respectively, it can be seen that the color changes were in noticeable range for washing time < 80 s at 47, 50, and 53°C, and 100 s at 47 and 50°C; whereas they were in well visible range when the washing time was 240 s at 47, 50, and 53°C, and 160 s at 53°C. In general, the color changes were acceptable based on the visual inspection.

Chemical treatments have been studied for removing *Salmonella* from produce but these are not permitted in many countries and alternative disinfection options are needed (Weiss and Hammes, 2005; Jarvis et al., 2016; Wei et al., 2017). Moreover, consumers prefer non-chemical interventions such as thermal treatments. For example, dipping shredded iceberg lettuce in chlorinated water at 47°C for 3 min could reduce the population of naturally occurring microorganisms by ~3 log CFU/g or at 50°C whether chlorinated or not (Delaquis et al., 1999; Li et al., 2001). Warm water washing reduces total numbers of aerobic bacteria, pseudomonads and Enterobacteriaceae on iceberg lettuce and mild heat significantly increased the efficacy of washing compared with cold water (Baur et al., 2005). There have been only a few studies addressing thermal inactivation of *Salmonella* on strawberries or other fruit although chlorination has been studied, while the potential safety risk related to toxic by-products formed by high levels of chlorine treatment has not been determined (Kaye et al., 2005; Mahmoud et al., 2007; Lu and Wu, 2010). Although the color change of washed strawberries were shown as decreases of lightness, redness and yellowness, leading to a slightly darker appearance more appealing to visual inspection, a greater bacterial reduction could be achieved to insure the safety at household level. Thus, we recommend that consumers wash strawberries with warm water before consumption.

### Parameter estimates of the primary models

**Figure 2:**
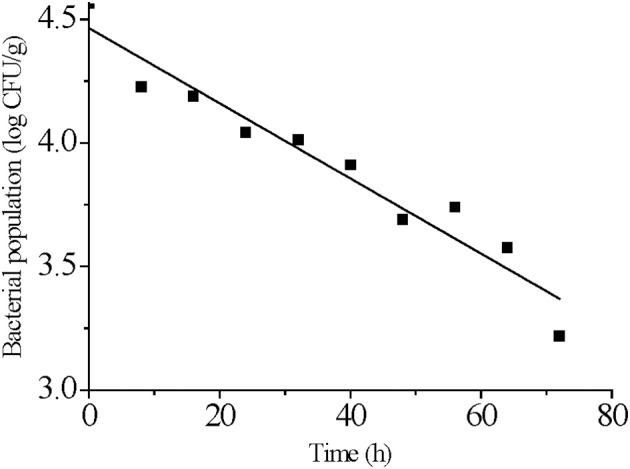
Log-linear model fitting for *S. Typhimurium* survival on strawberries at 4°C.

We used a log-linear model to fit *S*. Typhimurium survival at 4°C. The target *adj*-R^2^ (0.93) and RMSE (0.10) values were reached suggesting the log-linear model was reasonably accurate in describing the effect of time on the populations of *S*. Typhimurium on strawberries stored at 4°C (Figure 2 and Table 1). The log-linear model assumes a homogeneous bacterial die-off rate and therefore a linear curve was applied (Li et al., 2013). A mechanistic explanation for this is that bacterial death is the result of inactivation of a critical enzyme and this is governed by first-order inactivation kinetics (van Boekel, 2002). However, due to the heterogeneity between bacterial cells, differing probabilities of dying would make the survival curve non-linear.

**Table 1:** Parameter estimates and statistical analysis of the log-linear model fitted to *S. Typhimurium* survival curves at 4°C.

	**log *N_0_*^a^**	**D^b^**	***adj*-R^2^**	**RMSE^c^**
Log-linear model	4.47	65.74	0.93	0.10

a *initial bacterial population (log CFU/g)*.

b *decimal reduction time (s)*.

c *root mean square error*.

**Figure 3:**
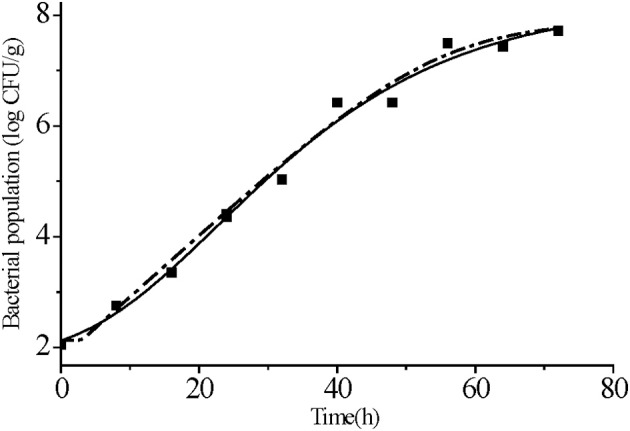
Primary models fitting for *S. Typhimurium* growth on strawberries at 25°C. Data points represent the mean values of the observed bacterial growth data at different times. Modified Gompertz model (solid line) and Huang model (dotted line).

Primary models that fit *S*. Typhimurium growth at 25°C indicated that both the modified Gompertz and Huang models agreed reasonably well with the observed data. The same *adj*-R^2^ (0.98) and RMSE (0.26) values for both these models indicated that they were adequate in describing the growth patterns (Figure 3 and Table 2). The AIC is a valuable tool for model selection and better models give lower AIC values (Ding et al., 2011). We found that the AIC statistic was slightly better for modified Gompertz model than for Huang (−7.26 *vs*. −7.05). Accordingly, the modified Gompertz model was chosen.

**Table 2:** Parameter estimates and statistical analysis of the primary models fitted to *S. Typhimurium* growth curves at 25°C.

	**log *N_0_*^a^**	**λ ^b^**	**log *N_max_*^c^**	**μ_max_^d^**	***adj*-R^2^**	**RMSE^e^**	**AIC^f^**
Huang model	2.13	3.01	7.90	0.11	0.98	0.26	−7.05
Modified gompertz model	1.95	3.36	8.31	0.12	0.98	0.26	−7.26

a *initial bacterial population (log CFU/g)*.

b *lag time (h)*.

c *maximum bacterial population (log CFU/g)*.

d *specific growth rate (h^−1^)*.

e *root mean square error*.

f *Akaike information criterion*.

**Figure 4:**
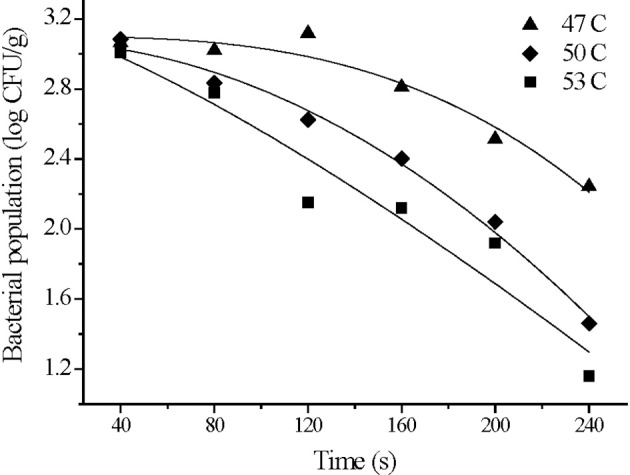
Weibull model fitting for *S. Typhimurium* survival on strawberries washed at 47, 50, and 53°C.

The survival curves of *S*. Typhimurium on strawberries washed at 47, 50, and 53°C fit the Weibull model. The high *adj*-R^2^ (≥0.90) and small RMSE (≤0.22) values indicated that this model was a good descriptor of bacterial behavior under mild heat treatments. The values of the shape parameter (*n*) at all three temperatures were>1 and agrees with the concave downward survival curves. These suggested that the bacterial inactivation rate increased with washing time. A shoulder was observed at 47°C indicating that the bacteria were less resistant at higher temperatures (Figure 4 and Table 3). The Weibull model assumes that the heat resistance of bacterial cells in a population follows a Weibull distribution and more accurately describes the shoulder effect or concave downward or upward curves (Peleg and Cole, 1998). The Weibull model is mathematically simple for fitting survival curves under thermal conditions. It is also more flexible than traditional first order models in describing the inactivation kinetics of food borne pathogens at 60°C (Buzrul and Alpas, 2007). The Weibull model has provided better fits than the log-linear model in determining the effectiveness of post-packaging hot water thermal treatment on survival of *Listeria innocua* in fully cooked chicken products (Li et al., 2013).

**Figure 5:**
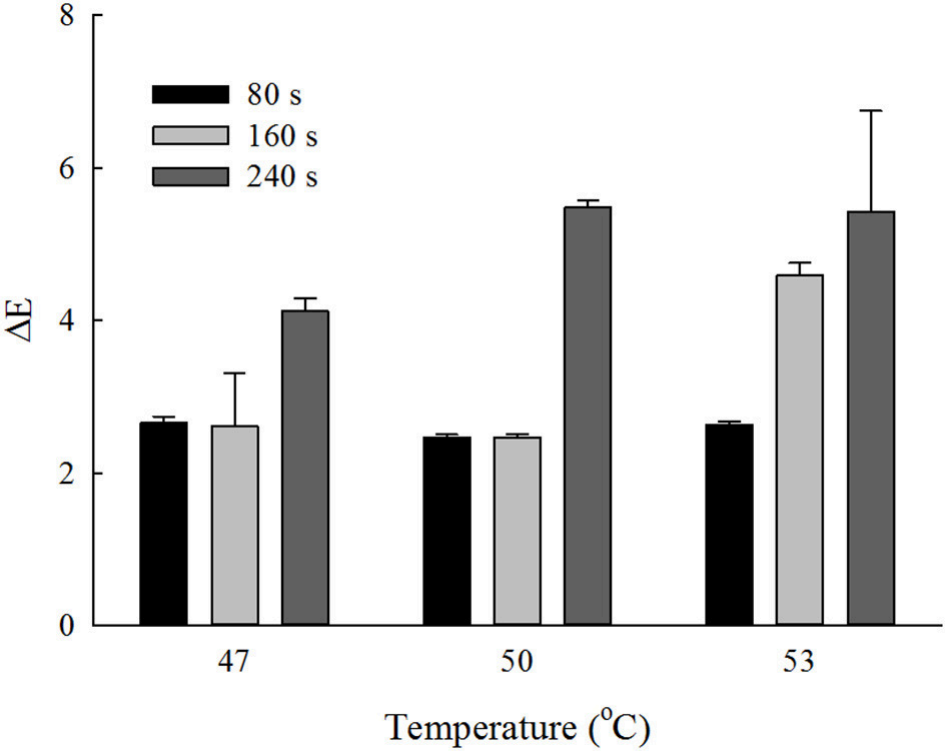
Total color change (Δ*E*) obtained at different temperature-time wash treatments of strawberries.

**Table 3:** Parameter estimates and statistical analysis of the Weibull model fitted to *S. Typhimurium* survival curves on strawberries washed at 47, 50, and 53°C.

**Models**	***B_*f*_***	***A_*f*_***
Modified Gompertz model	0.90	1.12
Log-linear model	0.88	1.14
ln *k* = 7.6–0.14^*^T	0.93	1.11
ln *n* = −93.79+1.65^*^T	0.89	1.10

### Influence of wash temperature on the weibull model parameters

**Figure 6:**
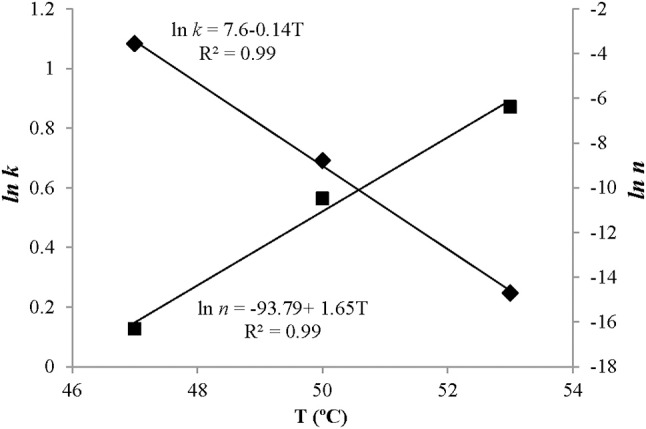
Effect of washing temperatures (T) on the kinetic parameters of the Weibull model. Points represent observed values and solid lines represent model fitting. ■, *ln n*; ♦, *ln k*.

The temperature dependence of the scale parameter *k* and shape parameter *n* agreed well with observed data. We found a close linear relationship between treatment temperatures and ln *k* and ln *n* with *R*^2^-values near 0.99 (Figure 6). In addition, the RMSEs of the model were 0.02 and 0.70 indicating acceptable agreement between predictions and observations.

### Model validation and its application in microbial risk assessment

Validation is a necessary step to evaluate the predictive ability a model, especially regarding *B*_*f*_ and *A*_*f*_ (Ross, 1996; Ding et al., 2011). We found that the *B*_*f*_ values of the modified Gompertz and ln *k* models fell within an acceptable range of 0.9–1.05. The log-linear and ln *n* models were also within the acceptable range of 0.7–0.9 or 1.06–1.15, respectively (Ross, 1999). The *A*_*f*_ takes the average distance between every point and the line of equivalence as a measure of how close, on average, the predictions are to the observations (Te-Giffel and Zwietering, 1999). The larger the value, the less accurate is the average estimate. Even without a structural deviation i.e., when *B*_*f*_ = 1, the accuracy factor is still functional (Ross, 1996). We found that the *A*_*f*_ values of the modified Gompertz model, log-linear model, ln *k* and ln *n* were 1.12, 1.14, 1.11, 1.10, respectively, indicating the predictions differ from the observations by 12, 14, 11, and 10%, respectively (Table 4). The acceptable *A*_*f*_ value for a single variable should be no >1.15 (Ross et al., 2000). Therefore, the *A*_*f*_ values we developed were within the acceptable range. Overall, the predictive models generated in this study were reasonable and can provide satisfactory predictions for *S*. Typhimurium growth and survival on fresh strawberries under different storage and wash temperatures.

**Table 4:** Validation indices calculated for the models used in this study.

**Temp (°C)**	**log *N_0_*^a^**	***k*^b^**	***n*^c^**	***adj*-R^2^**	**RMSE^d^**
47	3.09	8.24E-8	2.95	0.93	0.09
50	3.07	2.80E-5	1.99	0.98	0.07
53	3.17	1.69E-3	1.28	0.90	0.22

a *initial bacterial population (log CFU/g)*.

b *scale parameter*.

c *shape parameter*.

d *root mean square error*.

It is worth noting that the models developed in this study were based on challenge tests. Challenge test has been used as a useful method to characterize the microbial growth, survival and inactivation kinetic parameters, while the information collected can only applied for the specific product in those specific conditions, and most the models do not deal with individual variability between the cells as they are developed in a population level, using high inoculum level (Ross and McMeekin, 2003). Besides the individual cell variability, strain variability and complex food environment were also the challenges for the predictive microbiology models used in the risk assessment (Koutsoumanis et al., 2016). However, even though very detailed and specific predictive models have been studied in nowadays, the models used in published microbial quantitative microbial assessments remain simple compared to those developed in the predictive microbiology domain (Pouillot and Lubran, 2011). Moreover, Francois et al. (2006) investigated the impact of individual cell variability on the exposure assessment of *Listeria monocytogenes*, showing that the individual cell lag phase variability has important consequences when studying specific growth cases, but when performing more general exposure assessment studies, the variability between the individual cell lag phases is too limited to have a major impact on the total exposure assessment. Therefore, the predictive models embedded in a risk model should be compared and then selected according to the impact on the final risk estimate, in addition to the ability to fit the microbiological dataset.

## Conclusions

In this study, the survival of *S*. Typhimurium on strawberries was determined during storage under refrigeration. Bacteria growth was rapid on strawberries stored at room temperature. The bacterial inactivation effects of mild heat washing were significant, especially at higher temperatures. These results indicated that refrigeration and mild heat washing could effectively reduce the health risk of *S*. Typhimurium on strawberries. These models can provide useful tools in the development of *Salmonella* risk assessment model for strawberries.

## Author contributions

WenW designed the experiments, conducted the modeling, interpreted the results, and drafted the manuscript. YZ revised the manuscript. XX conducted the sensory analysis test and revised the manuscript. HY designed the sensory analysis experiment, interpret the results, and revised the manuscript. YL conducted the microbial experiments. GY, QW, and WeiW provided advices on results interpretation. All authors read and approved the final manuscript.

### Conflict of interest statement

The authors declare that the research was conducted in the absence of any commercial or financial relationships that could be construed as a potential conflict of interest.
